# Supplementary material for: Neutrophil-derived catecholamines mediate negative stress effects on bone
Source: Nat Commun. 2023 Jun 5;14:3262. doi: 10.1038/s41467-023-38616-0 (PMC10241819; doi:10.1038/s41467-023-38616-0)
Supplement: Supplementary file 1 — Supplementary Information [file 41467_2023_38616_MOESM1_ESM.pdf]

# Supplementary information for:

# Neutrophil-derived catecholamines mediate negative stress effects on bone

**Miriam E. A. Tschaffon-Müller[1, #], Elena Kempter[2, #], Lena Steppe[1], Sandra Kupfer[2], Melanie R. Kuhn[1], Florian Gebhard[3], Carlos Pankratz[3], Miriam Kalbitz[3,4], Konrad Schütze[3], Harald Gündel[5], Nele Kaleck[5], Gudrun Strauß[6], Jean Vacher[7], Hiroshi Ichinose[8], Katja Weimer[5], Anita Ignatius[1], Melanie Haffner-Luntzer[1, $], Stefan O. Reber[2, $, *]**

[1]Institute of Orthopaedic Research and Biomechanics, Ulm University Medical Center, Ulm, Germany.
[2]Laboratory for Molecular Psychosomatics, Department of Psychosomatic Medicine and Psychotherapy, Ulm University Medical Center, Ulm, Germany.
[3]Department of Orthopedic Trauma, Hand-, Plastic- and Reconstructive Surgery, Ulm University Medical Center, Ulm, Germany.
[4]Department of Trauma and Orthopedic Surgery, University Hospital Erlangen, Friedrich-Alexander University Erlangen-Nuremberg, Erlangen, Germany.
[5]Department of Psychosomatic Medicine and Psychotherapy, Ulm University Medical Center, Ulm, Germany.
[6]Department of Pediatrics and Adolescent Medicine, Ulm University Medical Center, Ulm, Germany
[7]Department of Medicine, Institut de Recherches Cliniques de Montréal, Montréal, Québec, Canada.
[8]School of Life Science and Technology, Tokyo Institute of Technology, Yokohama, Japan.

[#] *Both first authors contributed equally to this work*

*$ Both last authors contributed equally to this work*

** Corresponding author and reprint requests:*

Prof. Dr. Stefan O. Reber, Laboratory for Molecular Psychosomatics, Department of Psychosomatic Medicine and Psychotherapy, Ulm University Medical Center, N24, Albert-Einstein-Allee 11, 89081 Ulm, Germany, Phone: 0049 731 50061943, Fax: 0049 731 50061802, mailto: stefan.reber@uni-ulm.de

## Supplementary Figures and Supplementary Legends:

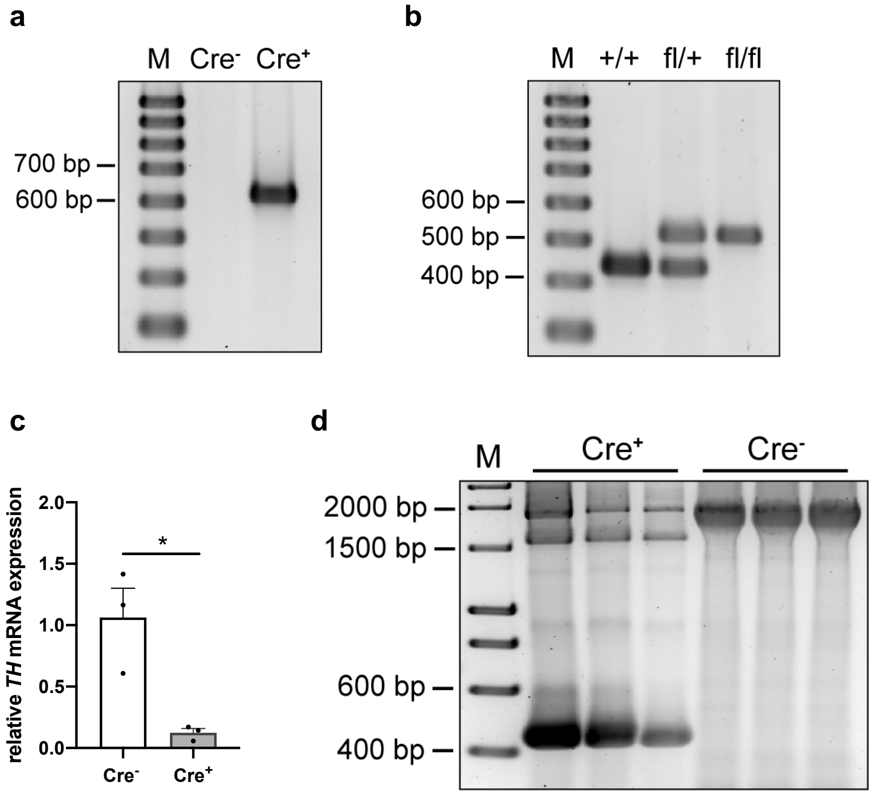


**Supplementary Figure 1. *Tyrosine hydroxylase* (*TH*) knockout verification in TH$^{flox/flox}$/ CD11b-Cre$^{+}$ mice (Set 5).** Gel documentation of (A) the CD11b-Cre and (B) wildtype (+) and floxed (fl) *TH* allele PCR product. (C) Relative *TH* mRNA expression in myeloid bone marrow cells isolated from TH$^{flox/flox}$/ CD11b-Cre$^{-}$ (TH$^{flox}$/Cre$^{-}$) and TH$^{flox/flox}$/ CD11b-Cre$^{+}$ (TH$^{flox}$/Cre$^{+}$) mice. *n* = 3; *P ≤ 0.05. (D) Detection of the floxed and the deleted TH allele PCR product in DNA isolated from myeloid bone marrow cells of TH$^{flox}$/Cre$^{+}$ and TH$^{flox}$/Cre$^{-}$ mice. M = marker. Data are displayed as mean + SEM including individual values. Source data, exact n-numbers, exact p-values and used statistical tests per panel are provided as a Source Data file.

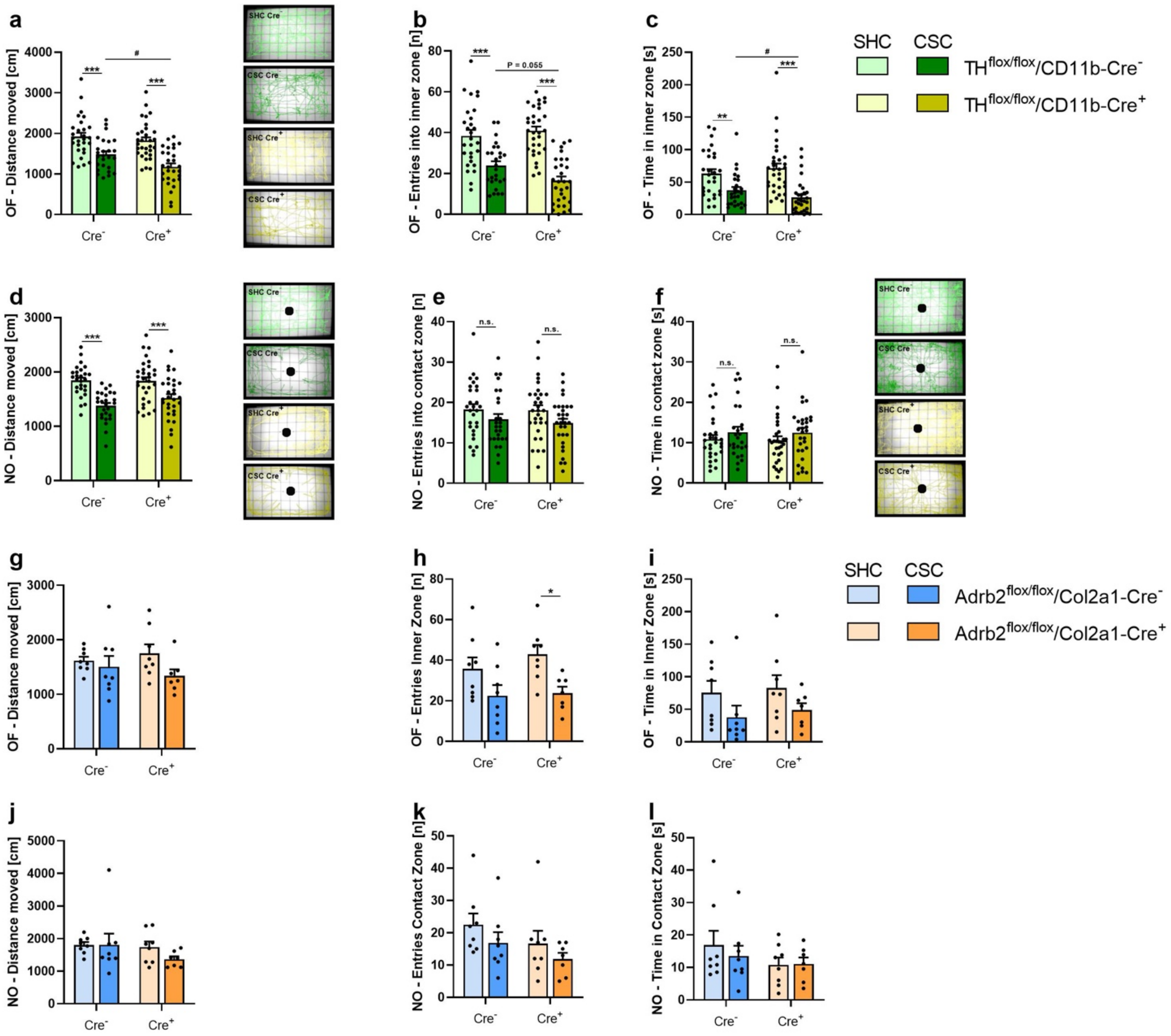


**Supplementary Figure 2. Effects of chronic subordinate colony housing (CSC) on anxiety-related behaviour assessed in the open field/novel object (OF/NO) test on Day 19 in TH$^{flox/flox}$/ CD11b-Cre (TH$^{flox}$/Cre) and Adrb2$^{flox/flox}$/Col2a1-Cre (Adrb2$^{flox}$/Cre) mice.** (A) Total distance moved, (B) entries into the inner zone and (C) time spent in the inner zone during open field (OF) exposure in TH$^{flox}$/Cre$^{-}$ and Cre$^{+}$ CSC and single housed control (SHC) mice. (D) Total distance moved, (E) entries into the contact zone and (F) time spent in the contact zone during novel object (NO) exposure in TH$^{flox}$/Cre$^{-}$ and Cre$^{+}$ mice. Track visualizations illustrate typical locomotion patterns of representative mice from each group. *n* = 27 - 31. (G) Total distance moved, (H) entries into the inner zone and (I) time spent in the inner zone during open field (OF) exposure in Adrb2$^{flox}$/Cre$^{-}$ and Adrb2$^{flox}$/Cre$^{+}$ SHC and CSC mice. (J) Total distance moved, (K) entries into the contact zone and (L) time spent in the contact zone during novel object (NO) exposure in Adrb2$^{flox}$/Cre$^{-}$ and Adrb2$^{flox}$/Cre$^{+}$ mice. *n* = 7 – 8. Data are presented as mean + SEM including individual values. *P ≤ 0.05, ***P ≤ 0.001 versus respective SHC condition; $^{\#}$P ≤ 0.05 versus respective Cre$^{-}$ group. n.s. = not significant. Source data, exact n-numbers, exact p-values and used statistical tests per panel are provided as a Source Data file.

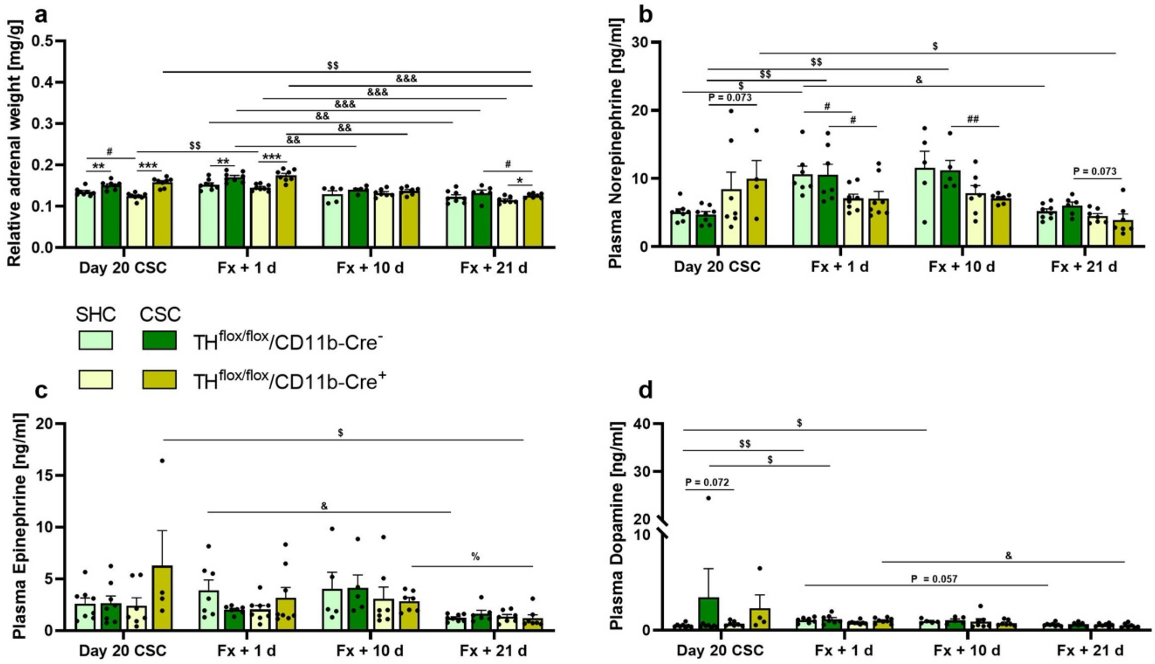


**Supplementary Figure 3. Effects of chronic subordinate colony housing (CSC) and subsequent femur osteotomy on adrenal weight and plasma catecholamine levels in $TH^{flox/flox}$/ CD11b-Cre ($TH^{flox}$/Cre) mice.** (A) Relative adrenal weight, (B) plasma norepinephrine (NE), C) plasma epinephrine (EPI) and D) plasma dopamine (DOP) levels in $TH^{flox/}$/$Cre^{-}$ and $TH^{flox/}$/$Cre^{+}$ CSC and single housed control (SHC) mice on Day 20 of CSC and 1 d, 10 d, and 21 d after subsequent femur osteotomy. *n* = 4 - 8. Data are presented as mean + SEM including individual values. *P ≤ 0.05, **P ≤ 0.01, ***P ≤ 0.001 versus respective SHC condition; $^{\#}$P ≤ 0.05, $^{\#\#}$P ≤ 0.01 versus respective $TH^{flox}$/$Cre^{-}$ group; $^{\$}$P ≤ 0.05, $^{\$\$}$P ≤ 0.01 versus respective group on d 20; $^{\&}$P ≤ 0.05, $^{\&\&}$P ≤ 0.01, $^{\&\&\&}$P ≤ 0.001 versus respective group at Fx + 1 d timepoint; $^{\%}$P ≤ 0.05 versus respective group at Fx + 10 d timepoint. n.s. = not significant. Source data, exact n-numbers, exact p-values and used statistical tests per panel are provided as a Source Data file.

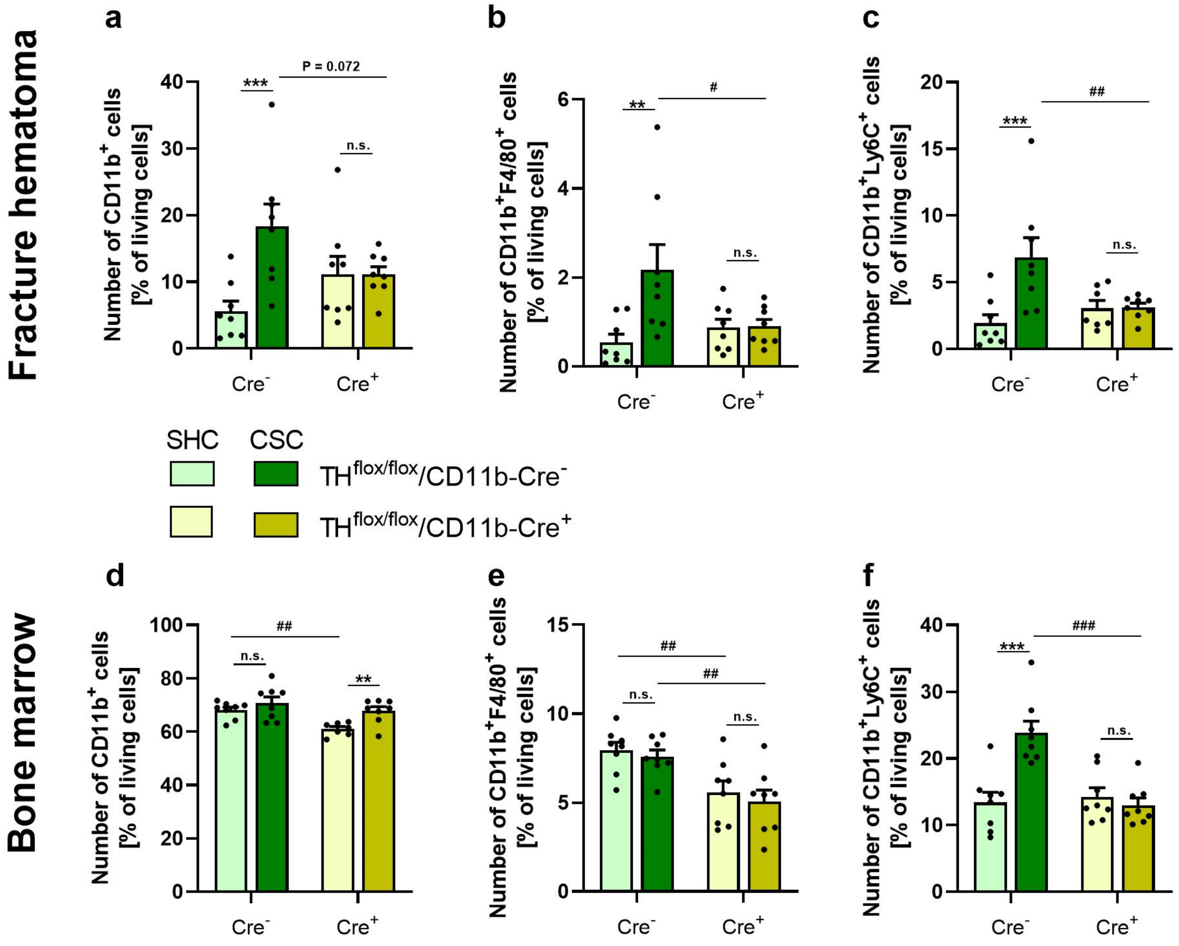


**Supplementary Figure 4. Effects of chronic subordinate colony housing (CSC) on cell composition in the fracture hematoma and the bone marrow 1 d after subsequent femur osteotomy in TH$^{flox/flox}$/ CD11b-Cre (TH$^{flox}$/Cre) mice.** Number of (A, D) CD11b$^{+}$ myeloid cells, (B, E) CD11b$^{+}$F4/80$^{+}$ macrophages and (C, F) CD11b$^{+}$Ly6C$^{+}$ monocytes in the (A-C) fracture hematoma and the (D-F) bone marrow of TH$^{flox}$/Cre$^{-}$ and Cre$^{+}$ mice exposed to CSC/ single housed control (SHC) conditions 1 d post-fracture. *n* = 7 - 8. Data are presented as mean + SEM including individual values. **P ≤ 0.01, ***P ≤ 0.001 versus respective SHC condition; $^{\#}$P ≤ 0.05, $^{\#\#}$P ≤ 0.01, $^{\#\#\#}$P ≤ 0.001 versus respective Cre$^{-}$ group. n.s. = not significant. Source data, exact n-numbers, exact p-values and used statistical tests per panel are provided as a Source Data file.

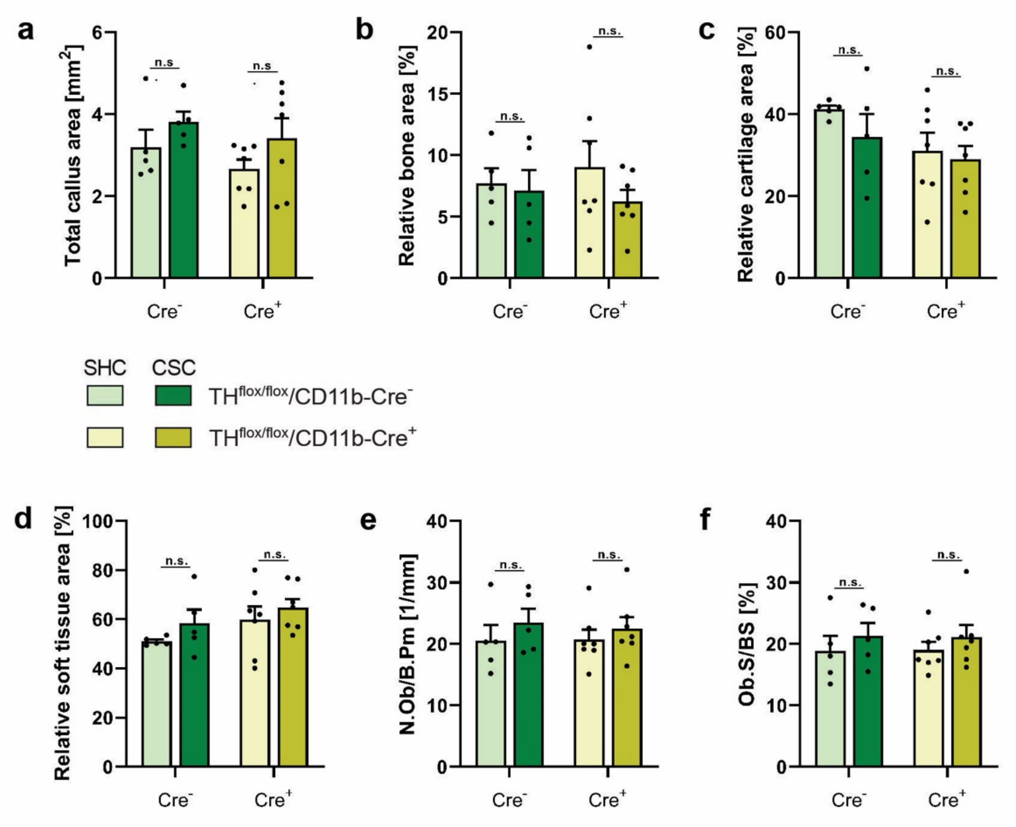


**Supplementary Figure 5. Effects of chronic subordinate colony housing (CSC) on fracture healing 10 d after subsequent femur osteotomy in TH$^{flox/flox}$/ CD11b-Cre (TH$^{flox}$/Cre) mice.** (A) total callus area, (B) relative bone area, (C) relative cartilage area, (D) relative soft tissue area, (E) number of osteoblasts (N.Ob) per bone perimeter (B.Pm) and (F) osteoblast surface (Ob.S) per BS in the fracture callus of TH$^{flox}$/Cre$^{-}$ and Cre$^{+}$ mice exposed to CSC/ single housed control (SHC) conditions 10 d post-fracture. *n* = 5 - 7. Data are presented as mean + SEM including individual values. n.s. = not significant. Source data, exact n-numbers, exact p-values and used statistical tests per panel are provided as a Source Data file.

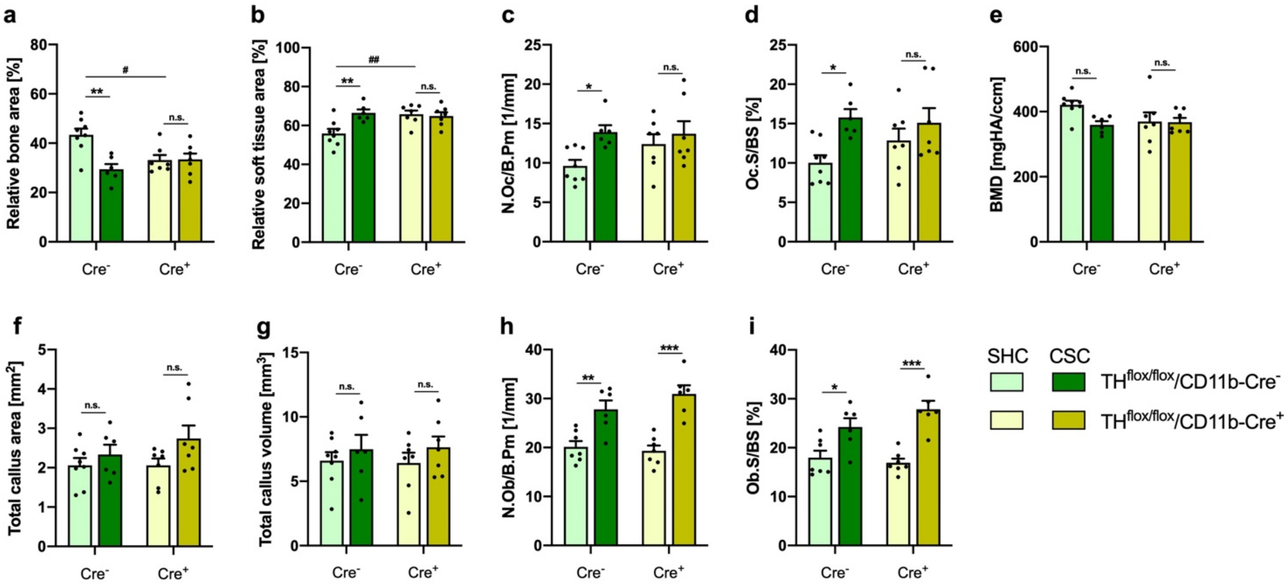


**Supplementary Figure 6. Effects of chronic subordinate colony housing (CSC) on fracture healing 21 d after subsequent femur osteotomy in $TH^{flox/flox}$/ CD11b-Cre ($TH^{flox}$/Cre) mice.** (A) Relative bone area, (B) relative soft tissue area, (C) number of osteoclasts (N.Oc) per bone perimeter (B.Pm), (D) osteoclast surface (Oc.S) per bone surface (BS), (E) bone mineral density (BMD), (F) total callus area, (G) total callus volume, (H) number of osteoblasts (N.Ob) per B. Pm and (I) osteoblast surface (Ob.S) per BS in the fracture callus of $TH^{flox}$/$Cre^-$ and $Cre^+$ mice exposed to CSC/ single housed control (SHC) conditions 21 d post-fracture. *n* = 6 - 8. Data are presented as mean + SEM including individual values. *P ≤ 0.05, **P ≤ 0.01, ***P ≤ 0.001 versus respective SHC condition; $^{\#}$P ≤ 0.05, $^{\#\#}$P ≤ 0.01 versus respective $Cre^-$ group. n.s. = not significant. Source data, exact n-numbers, exact p-values and used statistical tests per panel are provided as a Source Data file.

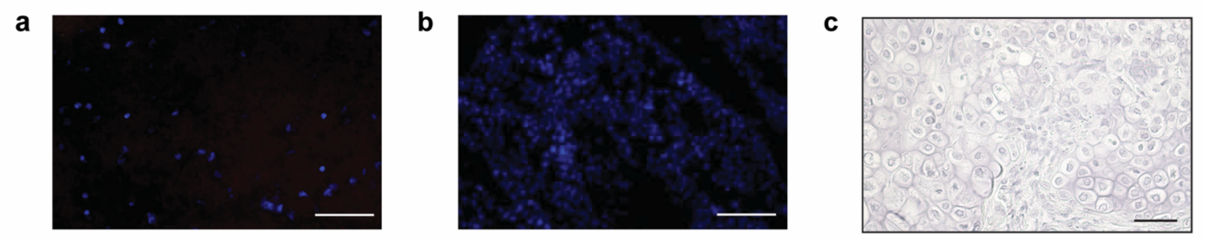


**Supplementary Figure 7. Negative controls for immunohistochemical stainings.** Respective negative control images for (A) immunofluorescent double staining of TH and CD16 in the fracture hematoma of patients, (B) immunofluorescent detection of TH in WT mice following 7 d of SHC/CSC exposure and (C) immunohistochemical detection of Runx2 in the fracture callus of $TH^{flox}/Cre^{-}$ und $Cre^{+}$ SHC and CSC mice 10 d post-fracture. Scale bars represent 50 µm. For every staining performed in this study, a corresponding negative control staining was performed in parallel.

## Tschaffon-Müller et al. 2023 Nat Comm Supplementary Figure 8

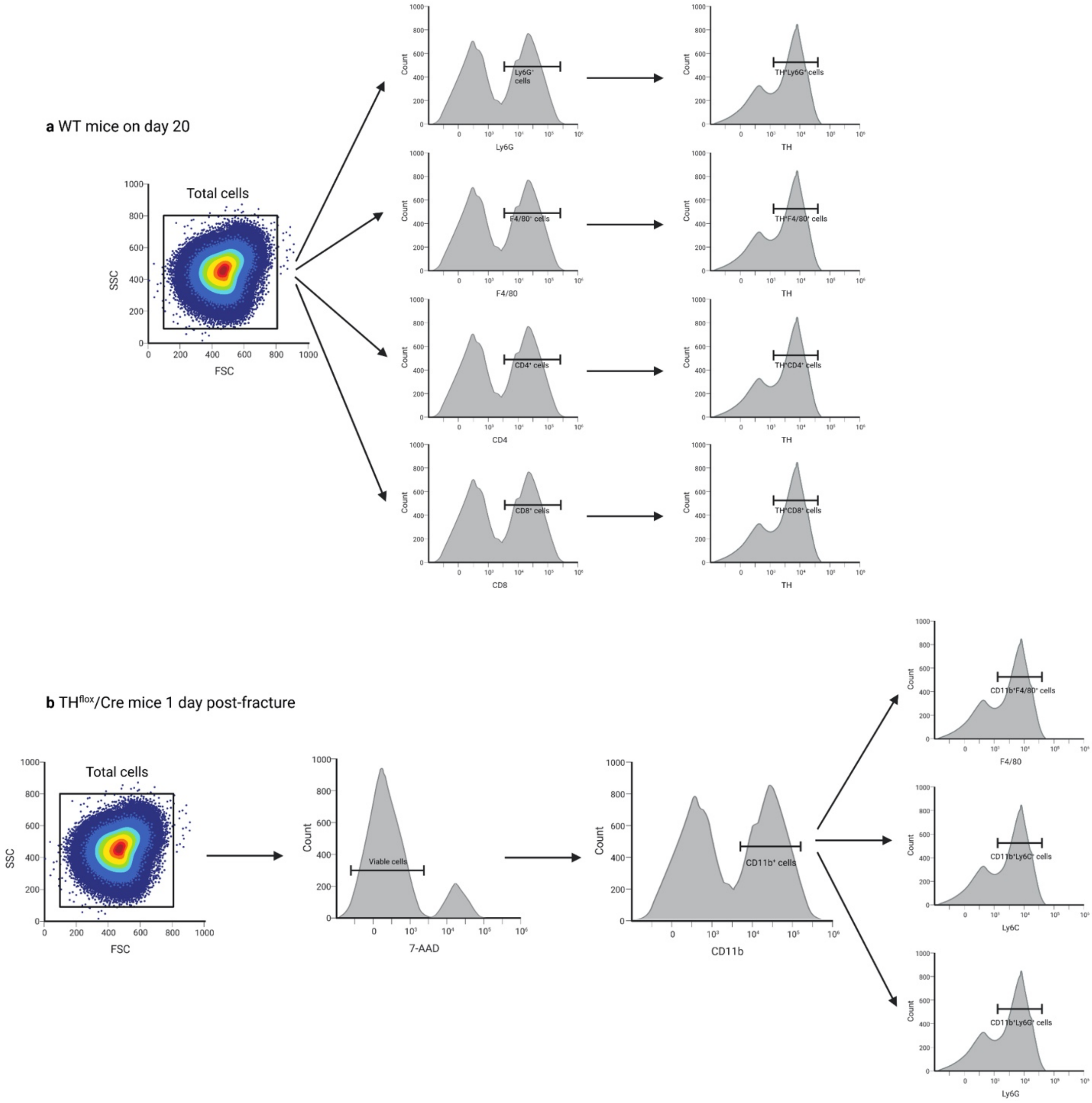


**Supplementary Figure 8. Gating strategies for flow cytometric analyses (created with BioRender.com).** (A) Gating strategy used for flow cytometry measurements of bone marrow (BM) cells of wildtype (WT) single housed control (SHC) and chronic subordinate colony housing (CSC) mice on Day 20 of the CSC paradigm. (B) Gating strategy used for flow cytometry measurements of fracture hematoma and BM cells of $TH^{flox}/Cre^{-}$ and $TH^{flox}/Cre^{+}$ SHC/CSC mice 1 d post femur osteotomy on Day 20 of the CSC paradigm.

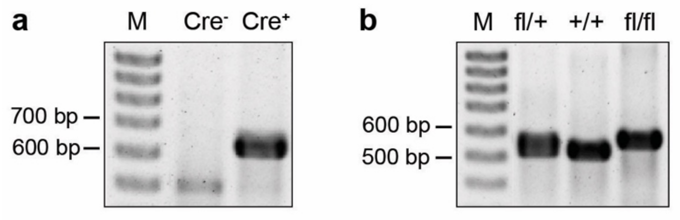


**Supplementary Figure 9. Genotyping of Adrb2$^{flox/flox}$/ Col2a1-Cre mice (Set 10).** Gel documentation of (A) the Col2a1-Cre and (B) wildtype (+) and floxed (fl) *Adrb2* allele PCR product.

## Supplementary Tables:

**Supplementary Table 1.** Spearman's correlation analysis between cytokines/growth factors in the plasma and psychosomatic parameters of upper ancle fracture patients. P-values between 0.05 and 0.10 are marked in italic. P-values below 0.05 are marked in bold.

| Spearman test | **BDNF** | **Eotaxin** | **HGF** | **IFNα** | **IFNγ** | **IL-1α** | **IL12p70** | **IL-18** | **IL1RA** | **IL-21** | **IL-27** | **IL-31** | **IL-5** | **IL-7** |
|---|---|---|---|---|---|---|---|---|---|---|---|---|---|---|
| **PHQ15 Som: r-value**<br>**p-value** | 0.305<br>0.232 | 0.207<br>0.422 | 0.268<br>0.295 | -0.078<br>0.764 | 0.248<br>0.334 | 0.055<br>0.834 | 0.377<br>0.136 | 0.129<br>0.619 | 0.097<br>0.710 | -0.122<br>0.637 | 0.186<br>0.472 | 0.055<br>0.833 | -0.090<br>0.728 | -0.221<br>0.390 |
| **GAD7 Anx: r-value**<br>**p-value** | 0.048<br>0.856 | 0.226<br>0.379 | 0.048<br>0.853 | 0.142<br>0.582 | *0.418*<br>*0.095* | -0.023<br>0.932 | *0.418*<br>*0.096* | 0.329<br>0.195 | 0.354<br>0.163 | -0.035<br>0.894 | 0.179<br>0.490 | 0.295<br>0.248 | -0.156<br>0.545 | -0.121<br>0.640 |
| **PHQ9 Depri: r-value**<br>**p-value** | 0.055<br>0.833 | 0.352<br>0.165 | 0.295<br>0.248 | 0.344<br>0.175 | 0.182<br>0.481 | -0.015<br>0.955 | *0.419*<br>*0.095* | 0.306<br>0.230 | 0.174<br>0.500 | -0.134<br>0.606 | 0.250<br>0.333 | -0.076<br>0.772 | 0.152<br>0.558 | -0.373<br>0.140 |
| **PHQS Stress: r-value**<br>**p-value** | -0.114<br>0.662 | *0.465*<br>*0.062* | 0.382<br>0.130 | **0.545**<br>**0.026** | 0.323<br>0.205 | -0.012<br>0.964 | *0.473*<br>*0.057* | *0.423*<br>*0.092* | 0.112<br>0.666 | -0.169<br>0.514 | 0.313<br>0.219 | -0.001<br>0.999 | 0.236<br>0.358 | -0.211<br>0.414 |
| **SF36 socfunc: r-value**<br>**p-value** | 0.136<br>0.610 | 0.185<br>0.481 | -0.184<br>0.482 | -0.195<br>0.454 | -0.102<br>0.701 | -0.301<br>0.242 | 0.008<br>0.983 | -0.297<br>0.251 | *-0.453*<br>*0.070* | *-0.437*<br>*0.080* | -0.337<br>0.188 | -0.370<br>0.145 | -0.134<br>0.607 | -0.172<br>0.512 |
| **SF36 pain: r-value**<br>**p-value** | 0.104<br>0.688 | -0.099<br>0.703 | -0.019<br>0.944 | -0.263<br>0.304 | **-0.606**<br>**0.012** | -0.324<br>0.203 | -0.397<br>0.116 | -0.408<br>0.105 | -0.090<br>0.729 | -0.179<br>0.486 | -0.371<br>0.144 | *-0.420*<br>*0.094* | *-0.428*<br>*0.088* | 0.094<br>0.718 |
| **CTQ sum: r-value**<br>**p-value** | *-0.472*<br>*0.0899* | 0.176<br>0.544 | 0.260<br>0.365 | 0.372<br>0.188 | 0.224<br>0.435 | -0.026<br>0.931 | 0.431<br>0.123 | 0.278<br>0.330 | -0.025<br>0.931 | 0.108<br>0.708 | 0.417<br>0.137 | 0.260<br>0.366 | 0.170<br>0.556 | -0.023<br>0.937 |

| Spearman test | **IP-10** | **LIF** | **MCP-1** | **MIP-1α** | **MIP-1β** | **NGFb** | **PDGF-BB** | **RANTES** | **SCF** | **SDF-1α** | **TNFα** | **VEGF-A** | **VEGF-D** |
|---|---|---|---|---|---|---|---|---|---|---|---|---|---|
| **PHQ15 Som: r-value**<br>**p-value** | 0.251<br>0.328 | -0.139<br>0.592 | -0.009<br>0.975 | 0.180<br>0.484 | *0.429*<br>*0.087* | 0.174<br>0.501 | 0.188<br>0.467 | 0.113<br>0.664 | 0.183<br>0.480 | 0.161<br>0.534 | 0.324<br>0.204 | 0.280<br>0.274 | 0.302<br>0.236 |
| **GAD7 Anx: r-value**<br>**p-value** | 0.200<br>0.437 | -0.023<br>0.929 | -0.010<br>0.971 | 0.021<br>0.937 | 0.252<br>0.327 | **0.513**<br>**0.037** | 0.303<br>0.234 | 0.394<br>0.118 | 0.249<br>0.333 | 0.223<br>0.387 | **0.500**<br>**0.043** | -0.062<br>0.812 | 0.353<br>0.163 |
| **PHQ9 Depri: r-value**<br>**p-value** | 0.289<br>0.258 | 0.045<br>0.862 | 0.082<br>0.754 | 0.330<br>0.194 | *0.440*<br>*0.078* | 0.346<br>0.173 | -0.077<br>0.768 | 0.140<br>0.591 | 0.256<br>0.319 | 0.174<br>0.502 | 0.398<br>0.114 | 0.051<br>0.845 | 0.245<br>0.340 |
| **PHQS Stress: r-value**<br>**p-value** | 0.266<br>0.300 | 0.068<br>0.793 | 0.119<br>0.648 | 0.265<br>0.300 | 0.310<br>0.225 | **0.521**<br>**0.034** | 0.082<br>0.753 | 0.196<br>0.448 | 0.350<br>0.168 | 0.185<br>0.475 | **0.491**<br>**0.047** | -0.188<br>0.467 | 0.574<br>0.018 |
| **SF36 socfunc: r-value**<br>**p-value** | -0.113<br>0.671 | -0.249<br>0.340 | 0.227<br>0.386 | -0.340<br>0.185 | -0.192<br>0.461 | 0.051<br>0.845 | -0.169<br>0.513 | -0.238<br>0.359 | -0.388<br>0.127 | -0.061<br>0.820 | -0.083<br>0.753 | 0.063<br>0.811 | -0.038<br>0.880 |
| **SF36 pain: r-value**<br>**p-value** | -0.279<br>0.276 | -0.251<br>0.328 | *-0.458*<br>*0.066* | -0.316<br>0.214 | -0.278<br>0.277 | **-0.694**<br>**0.003** | -0.081<br>0.754 | **-0.614**<br>**0.010** | -0.345<br>0.174 | -0.178<br>0.492 | **-0.521**<br>**0.034** | 0.034<br>0.898 | -0.368<br>0.145 |
| **CTQ sum: r-value**<br>**p-value** | 0.081<br>0.780 | 0.121<br>0.674 | 0.171<br>0.554 | *0.501*<br>*0.069* | 0.158<br>0.586 | 0.241<br>0.400 | 0.039<br>0.892 | 0.129<br>0.657 | 0.228<br>0.427 | -0.198<br>0.493 | 0.113<br>0.695 | 0.007<br>0.980 | 0.140<br>0.629 |

**Supplementary Table 2.** Primer sequences for mouse genotyping and knockout verification.

| gene construct | forward primer sequence (5' to 3') | reverse primer sequence (3' to 5') |
|---|---|---|
| CD11b-Cre | AATGCTTCTGTCCGTTTGC | CGGCAACACCATTTTTTCTG |
| TH flox | CATTTGCCCAGTTCTCCCAG | AGAGATGCAAGTCCAATGTC |
| TH KO | AGGCGTATCGCCAGCGCCGG | CCCCAGAGATGCAAGTCCAATGTC |
| Col2a1-Cre | GAG TGA TGA GGT TCG CAA GA | CTA CAC CAG AGA CGG |
| Adrb2 flox | CCAAAGTTGTTGCACGTCAC | GCACACGCCAAGGAGATTAT |

**Supplementary Table 3.** Antibodies for FACS analysis.

| antibody | company | cat. number | dilution |
|---|---|---|---|
| rat-anti-CD11b-APC-Cy7 | eBioscience | 47-0112-82 | 1:400 |
| rat-CD4-FITC | BioLegend | 100406 | 1:200 |
| rat-anti-CD8a-APC | eBioscience | 17-0081-81 | 1:200 |
| rat-anti-F4/80-FITC | eBioscience | 11-4801-82 | 1:50 |
| rat-anti-Ly6C-APC | BD Biosciences | 560595 | 1:200 |
| rat-anti-Ly6G-FITC | BioLegend | 127605 | 1:200 |
| rat-anti-Ly6G-V450 | BD Biosciences | 560603 | 1:400 |
| rat-anti-Tyrosine Hydroxylase-PE | Abcam | ab209921 | 1:200 |
| rat IgG2a-FITC | eBioscience | 11-4321-82 | 1:50 |
| rat IgG2a-V450 | BD Biosciences | 560377 | 1:400 |
| rat IgG2b-APC-eFl780 | eBioscience | 47-4031-82 | 1:400 |
| rat IgM-APC | BD Biosciences | 551486 | 1:200 |

**Supplementary Table 4.** Primer sequences for gene expression analysis.

| gene | forward primer sequence (5' to 3') | reverse primer sequence (3' to 5') |
|---|---|---|
| *Alpl* | GCTGATCATTCCCACGTTTT | GAGCCAGACCAAAGATGGAG |
| *B2m* | ATACGCCTGCAGAGTTAAGCA | TCACATGTCTCGATCCCAGT |
| *Cbfa1* | CCACCACTCACTACCACACG | CACTCTGGCTTTGGGAAGAG |
| *Nanog* | AAGGATGAAGTGCAAGCGGT | GGTGCTGAGCCCTTCTGAAT |
| *Sox2* | CAAAAACCGTGATGCCGACT | CGCCCTCAGGTTTTCTCTGT |
| *Sp7* | CCTTAACCCAGCTCCCTACC | ACCGCCTTGGGCTTATAGAC |
| *TH* | GCTTCTCTGACCAGGCGTAT | GGAATTGGCTCACCCTGCTT |

**Supplementary Table 5.** Patient characteristics. Body mass index (BMI; =body weight in kg/(height in m)$^{2)}$. Alcohol consumption: 1 = daily, 2 = 2-3x/week, 3 = 1x/week, 4 = 2-3x/month, 5 = 1x/months, 6 = less than 1x/months.

| Patient no. | age in years | gender | BMI | smoking | alcohol | diabetes |
|---|---|---|---|---|---|---|
| 1 | 30 | male | 31.6 | no | 6 | no |
| 2 | 37 | male | 34.3 | yes | 2 | no |
| 3 | 47 | male | 25.0 | yes | 3 | no |
| 4 | 65 | male | 27.8 | no | 4 | yes |
| 5 | 63 | female | 44.5 | no | 6 | yes |
| 6 | 68 | male | 28.7 | no | 2 | yes |
| 7 | 55 | female | 32.9 | yes | 4 | no |
| 8 | 48 | male | 25.3 | no | 3 | no |
| 9 | 32 | male | 23.2 | yes | 1 | no |
| 10 | 30 | female | 21.5 | yes | 5 | no |
| 11 | 43 | female | 40.6 | yes | 5 | no |
| 12 | 28 | male | 29.4 | no | 6 | no |
| 13 | 70 | female | 34.1 | no | 6 | no |
| 14 | 21 | male | 24.5 | no | 2 | no |
| 15 | 39 | female | 42.5 | no | 6 | no |
| 16 | 20 | male | 24.3 | yes | 3 | no |
| 17 | 71 | male | 24.4 | no | 3 | no |
| 18 | 65 | female | 24.9 | no | 2 | no |
| 19 | 57 | female | 37.8 | no | 6 | no |
| 20 | 52 | female | 37.2 | yes | 3 | no |
