# Supplementary material for: Neutrophil-derived catecholamines mediate negative stress effects on bone
Source: Nat Commun. 2023 Jun 5;14:3262. doi: 10.1038/s41467-023-38616-0 (PMC10241819; doi:10.1038/s41467-023-38616-0)
Supplement: Supplementary file 3 — Reporting Summary [file 41467_2023_38616_MOESM3_ESM.pdf]

nature portfolio

Corresponding author(s): Stefan O. Reber

Last updated by author(s): Apr 21, 2023

Reporting Summary

Nature Portfolio wishes to improve the reproducibility of the work that we publish. This form provides structure for consistency and transparency in reporting. For further information on Nature Portfolio policies, see our Editorial Policies and the Editorial Policy Checklist.

Statistics

For all statistical analyses, confirm that the following items are present in the figure legend, table legend, main text, or Methods section.

n/a	Confirmed	
☐	☒	The exact sample size (*n*) for each experimental group/condition, given as a discrete number and unit of measurement
☐	☒	A statement on whether measurements were taken from distinct samples or whether the same sample was measured repeatedly
☐	☒	The statistical test(s) used AND whether they are one- or two-sided<br>*Only common tests should be described solely by name; describe more complex techniques in the Methods section.*
☐	☒	A description of all covariates tested
☐	☒	A description of any assumptions or corrections, such as tests of normality and adjustment for multiple comparisons
☐	☒	A full description of the statistical parameters including central tendency (e.g. means) or other basic estimates (e.g. regression coefficient) AND variation (e.g. standard deviation) or associated estimates of uncertainty (e.g. confidence intervals)
☐	☒	For null hypothesis testing, the test statistic (e.g. *F*, *t*, *r*) with confidence intervals, effect sizes, degrees of freedom and *P* value noted<br>*Give P values as exact values whenever suitable.*
☒	☐	For Bayesian analysis, information on the choice of priors and Markov chain Monte Carlo settings
☒	☐	For hierarchical and complex designs, identification of the appropriate level for tests and full reporting of outcomes
☐	☒	Estimates of effect sizes (e.g. Cohen's *d*, Pearson's *r*), indicating how they were calculated

Our web collection on statistics for biologists contains articles on many of the points above.

Software and code

Policy information about availability of computer code

Data collection	CTAn (Version 10, Bruker, Massachusetts, USA)
Data analysis	EthoVision XT (Version 9, Noldus Information Technology, Wageningen, Netherlands); CTVol (Version 10, Bruker, Massachusetts, USA); GraphPad Prism (version 9.3.1, GraphPad Software, LCC); Leica Application Suite X software (Leica, Wetzlar, Germany); BD FACS DIVA (BD Bioscience)

For manuscripts utilizing custom algorithms or software that are central to the research but not yet described in published literature, software must be made available to editors and reviewers. We strongly encourage code deposition in a community repository (e.g. GitHub). See the Nature Portfolio guidelines for submitting code & software for further information.

Data

Policy information about availability of data

All manuscripts must include a data availability statement. This statement should provide the following information, where applicable:

- Accession codes, unique identifiers, or web links for publicly available datasets
- A description of any restrictions on data availability
- For clinical datasets or third party data, please ensure that the statement adheres to our policy

All data are provided in the source data file.

Research involving human participants, their data, or biological material

Policy information about studies with human participants or human data. See also policy information about sex, gender (identity/presentation), and sexual orientation and race, ethnicity and racism.

Reporting on sex and gender

Findings were generated in a group of men and women after written consent has been obtained. Sex of the participants was obtained from the medical records, gender was self-reported. In our study, sex and gender matched for all participants.

Reporting on race, ethnicity, or other socially relevant groupings

We did not obtain information regarding race or ethnicity from our patients.

Population characteristics

Supplementary table 5 summarized important patient characteristics (age, gender, BMI, smoking, alcohol consumption) from the 20 patients who were willing to fill-in the additional questionaires at the time point of surgery.

Patient no. age in years gender BMI smoking alcohol diabetes
1 30 male 31.6 no 6 no
2 37 male 34.3 yes 2 no
3 47 male 25.0 yes 3 no
4 65 male 27.8 no 4 yes
5 63 female 44.5 no 6 yes
6 68 male 28.7 no 2 yes
7 55 female 32.9 yes 4 no
8 48 male 25.3 no 3 no
9 32 male 23.2 yes 1 no
10 30 female 21.5 yes 5 no
11 43 female 40.6 yes 5 no
12 28 male 29.4 no 6 no
13 70 female 34.1 no 6 no
14 21 male 24.5 no 2 no
15 39 female 42.5 no 6 no
16 20 male 24.3 yes 3 no
17 71 male 24.4 no 3 no
18 65 female 24.9 no 2 no
19 57 female 37.8 no 6 no
20 52 female 37.2 yes 3 no

Recruitment

36 patients with upper ankle fractures treated surgically at the Department of Orthopedic Trauma, Hand-, Plastic- and Reconstructive Surgery at Ulm University Medical Centre between August 2018 and August 2021 were included. Exclusion criteria were: polytrauma, pregnancy, bone diseases except primary osteoporosis, intake of bisphosphonates or parathyroid hormone, rheumatoid arthritis, open fractures of grades 3 or 4 according to Tscherne and Oestern, hepatic or renal insufficiency, cancer, intake of steroids or immunosuppressive medication, chemotherapy in the last 3 months and artificial ventilation following surgery. Patients were recruited based on their willingness to participate in the study.

Ethics oversight

The clinical study was approved by the Ethical Committee of the Ulm University Medical Centre and conducted in accordance with the declaration of Helsinki (approval number 219/18)

Note that full information on the approval of the study protocol must also be provided in the manuscript.

Field-specific reporting

Please select the one below that is the best fit for your research. If you are not sure, read the appropriate sections before making your selection.

☒ Life sciences ☐ Behavioural & social sciences ☐ Ecological, evolutionary & environmental sciences

For a reference copy of the document with all sections, see nature.com/documents/nr-reporting-summary-flat.pdf

Life sciences study design

All studies must disclose on these points even when the disclosure is negative.

Sample size

Sample size calculation was done prior to the study by using G power software (Universität Düsseldorf, Germany).

Data exclusions

Outliers in normally distributed data sets were identified by Grubbs test and excluded from further analysis (one outlier was removed in the CSC group in the number of TH+Ly6G+ cells (Fig 2N) and in the SHC group in the number of TH+CD4+ cells (Fig 2P); one outlier was removed in the SHC THflox/Cre+ group in the number of CD11b+Ly6G+ cells in both the hematoma (Fig 4B) and in the number of CD11b+ cells in the bone marrow (SupFig 4D), respectively; one outlier was removed in the CSC THflox/Cre+ group in the distance moved during OF conditions (SupFig 2A); one outlier was removed in the SHC THflox/Cre- group in the distance moved during NO conditions (SupFig 2D) and the entries into the contact zone (SupFig 2E); one outlier was removed in the tibia length of CSC mice on Day 8 (Fig 2K); one outlier was removed in the trabecular

	thickness of CSC mice on Day 20 + 21 d of single housing (SH; Fig 2S); one outlier was removed in the SHC Adrb2flox/Cre+ group in the trabecular tissue mineral density (Tb. TMD; Fig 6F)).
Replication	All data is this study are obtained from independent mice and therefore are not replicates. However, we could verify data obtained from previous studies using the CSC mice, indicating reproducibility and reliability of that model.
Randomization	Mice were ramdonly divided into the different groups according to their genotype. For the clinical study, randomization is not relevant as this is an observational study.
Blinding	For the mouse study, investigators were not blinded. For the clinical study, investigators were blinded as patient characteristics were pseudomized during the trial. Unblinding was done at the end of data collection and analysis.

Reporting for specific materials, systems and methods

We require information from authors about some types of materials, experimental systems and methods used in many studies. Here, indicate whether each material, system or method listed is relevant to your study. If you are not sure if a list item applies to your research, read the appropriate section before selecting a response.

Materials & experimental systems

n/a	Involved in the study
☐	☒ Antibodies
☐	☒ Eukaryotic cell lines
☒	☐ Palaeontology and archaeology
☐	☒ Animals and other organisms
☒	☐ Clinical data
☒	☐ Dual use research of concern
☒	☐ Plants

Methods

n/a	Involved in the study
☒	☐ ChIP-seq
☐	☒ Flow cytometry
☒	☐ MRI-based neuroimaging

Antibodies

Antibodies used	rabbit anti-Runx2 (1:50, #8486, Cell Signaling, Danvers, Massachusetts, USA); rabbit-anti-Osteocalcin (1:200, #orb77248, Biorbyt, Cambridge, UK); biotin-XX-goat anti-rabbit (1:200, #B2770, Life technologies, Carlsbad, CA, USA); rabbit anti-human TH antibody (1:50, Merck #AB152); rat anti-CD31 (1:10, #DIA-310, Dianova); rabbit-anti-colX (1:200, #ABIN1077945, Antibodies Online); biotin-XX-goat anti-rabbit (1:200, #B2770, Life technologies, Carlsbad, CA, USA); rat-anti-CD11b-APC-Cy7 eBioscience 47-0112-82 1:400<br>rat-CD4-FITC BioLegend 100406 1:200<br>rat-anti-CD8a-APC eBioscience 17-0081-81 1:200<br>rat-anti-F4/80-FITC eBioscience 11-4801-82 1:50<br>rat-anti-Ly6C-APC BD Biosciences 560595 1:200<br>rat-anti-Ly6G-FITC BioLegend 127605 1:200<br>rat-anti-Ly6G-V450 BD Biosciences 560603 1:400<br>rat-anti-Tyrosine Hydroxylase-PE Abcam ab209921 1:200<br>rat IgG2a-FITC eBioscience 11-4321-82 1:50<br>rat IgG2a-V450 BD Biosciences 560377 1:400<br>rat IgG2b-APC-eFl780 eBioscience 47-4031-82 1:400<br>rat IgM-APC BD Biosciences 551486 1:200
Validation	All staining procedures involving antibodies were validated by using negative controls (species specific IgG).

Eukaryotic cell lines

Policy information about cell lines and Sex and Gender in Research

Cell line source(s)	ATDC5 cells (European Collection of Authenticated Cell Cultures (ECACC))
Authentication	None of the cell lines were authenticated.
Mycoplasma contamination	All cell lines tested negative for mycoplasma contamination.
Commonly misidentified lines (See ICLAC register)	none

Animals and other research organisms

Policy information about studies involving animals; ARRIVE guidelines recommended for reporting animal research, and Sex and Gender in Research

Laboratory animals	Male THflox/flox/CD11b-Cre+ mice (referred to as THflox/Cre+ mice) of Set 1-4 with a specific TH knockout in myeloid cells were generated by crossing female THflox/flox mice (Thtm1.1Ich), kindly provided by Prof. Dr. Ichinose, Tokyo Institute of Technology, Japan33 with male CD11b-Cre mice (Tg(ITGAM-cre)2781Gkl), kindly provided by Dr. Vacher, Institut de Recherches Cliniques de Montréal, Québec, Canada.34 Due to location of the CD11b-Cre construct on the Y chromosome, male THflox/flox/CD11b-Cre- control mice (referred to as THflox/Cre- mice) of Set 1-4 were generated by backcrossing female THflox/flox/CD11b-Cre mice with male THflox/flox mice. Verification of a successful TH KO in CD11b+ cells from THflox/Cre+ but not THflox/Cre- mice was done in myeloid BM cells isolated from additional 3 mice per group (Set 5). Male C57BL/6N (wild type; WT) mice of Set 6 weighing 19-22 g were obtained from Charles River (Sulzfeld, Germany) to assess TH expression in various leukocyte subpopulations isolated from bone marrow. For generation of conditioned medium used in the in vitro transdifferentiation assay CD11b+ BM cells were isolated from another group of WT mice exposed to 19 d of CSC or SHC (Set 7). Additional sets of mice were subjected to 7 d of CSC (Set 8) and 19 d of CSC followed by 21 d of single housing (SH; Set 9) to investigate acute and long-term effects of our stress model on bone. Furthermore, Adrb2flox/flox/Col2a1-Cre+ mice and their Cre- littermates (referred as Adrb2flox/Cre+ and Adrb2flox/Cre- mice, Set 10) were generated by crossing Adrb2flox/flox mice (Adrb2tm1Kry), generously provided by Prof. Karsenty, Department of Genetics & Development, Columbia University Medical Center, New York, USA35, with Col2a1-Cre mice (B6;SJL-Tg(Col2a1-cre)1Bhr/J) (JAX stock #00355436). Genotyping of THflox/Cre and Adrb2flox/Cre mice was performed using primer pairs listed in SupTab 2. Male CD-1 mice (30-35 g, Charles River, Sulzfeld, Germany) were used as dominant aggressors in the CSC paradigm.
Wild animals	The study did not involve wild animals.
Reporting on sex	Only male mice were used for the study because the CD11b-Cre transgen is located on the Y chromosome.
Field-collected samples	The study did not involve field-collected samples.
Ethics oversight	All experiments were approved by the Committee on Animal Health and Care of the local government: Regierungspräsidium Tübingen (TVAs 1195, 1216, 1219, 1267 and 1437, o135-7) and performed according to international guidelines on the ethical use of animals.

Note that full information on the approval of the study protocol must also be provided in the manuscript.

Flow Cytometry

Plots

Confirm that:

☒ The axis labels state the marker and fluorochrome used (e.g. CD4-FITC).

☒ The axis scales are clearly visible. Include numbers along axes only for bottom left plot of group (a 'group' is an analysis of identical markers).

☒ All plots are contour plots with outliers or pseudocolor plots.

☒ A numerical value for number of cells or percentage (with statistics) is provided.

Methodology

Sample preparation	Bone marrow (BM) and fracture hematoma cells were isolated from THflox/Cre+ and Cre- mice euthanized 1 d post-fracture (Set 2) and WT mice euthanized on Day 20 of the CSC paradigm (Set 6). Cells were incubated for 30 min with fluorescent labelled antibodies listed in SupTab 2. Host-specific isotype controls were used as negative control. Cells from fractured THflox/Cre+ and Cre- mice were additionally stained with 7-AAD (1:200).
Instrument	Flow cytometric analysis was performed using LSRII flow cytometer (BD Bioscience)
Software	Flow cytometric analysis was performed using BD FACS Diva (BD Bioscience) software.
Cell population abundance	The percentage of the cell populations are described in the manuscript. We did not sort the cells, we only analyzed them.
Gating strategy	The gating strategies used for the analysis of the flow cytometric measurements are shown in SupFig 8. SSC/FSC gates were set to include all cells except cell debris and duplicates (always the same for all expeirments). Gating for negative vs. positive cells was done according to the negative control IgG used in the study.

☒ Tick this box to confirm that a figure exemplifying the gating strategy is provided in the Supplementary Information.
